# Supplementary material for: Real-world clinical practice of Diabetic Foot Ulcer prevention and care in Singapore: A qualitative inquiry with healthcare professionals
Source: PLoS One. 2025 Aug 11;20(8):e0328637. doi: 10.1371/journal.pone.0328637 (PMC12338812; doi:10.1371/journal.pone.0328637)
Supplement: S1 Appendix — (PDF) [file pone.0328637.s001.pdf]

S1_Appendix: SRQR

Guide questions/description	Section
Title - Concise description of the nature and topic of the study Identifying the study as qualitative or indicating the approach (e.g., ethnography, grounded theory) or data collection methods (e.g., interview, focus group) is recommended	Title
Abstract - Summary of key elements of the study using the abstract format of the intended publication; typically includes background, purpose, methods, results, and conclusions	Abstract
Introduction	
Problem formulation - Description and significance of the problem/phenomenon studied; review of relevant theory and empirical work; problem statement	Introduction
Purpose or research question - Purpose of the study and specific objectives or questions	Introduction
Methods	
Qualitative approach and research paradigm - Qualitative approach (e.g., ethnography, grounded theory, case study, phenomenology, narrative research) and guiding theory if appropriate; identifying the research paradigm (e.g., postpositivist, constructivist/ interpretivist) is also recommended; rationale**	Methods>study design
Researcher characteristics and reflexivity - Researchers' characteristics that may influence the research, including personal attributes, qualifications/experience, relationship with participants, assumptions, and/or presuppositions; potential or actual interaction between researchers' characteristics and the research questions, approach, methods, results, and/or transferability	Methods>data collection
Context - Setting/site and salient contextual factors; rationale**	Methods>data collection
Sampling strategy - How and why research participants, documents, or events were selected; criteria for deciding when no further sampling was necessary (e.g., sampling saturation); rationale**	Methods>data collection
Ethical issues pertaining to human subjects - Documentation of approval by an appropriate ethics review board and participant consent, or explanation for lack thereof; other confidentiality and data security issues	Methods>ethical approval
Data collection methods - Types of data collected; details of data collection procedures including (as appropriate) start and stop dates of data collection and analysis, iterative process, triangulation of sources/methods, and modification of procedures in response to evolving study findings; rationale**	Methods>data collection
Data collection instruments and technologies - Description of instruments (e.g., interview guides, questionnaires) and devices (e.g., audio recorders) used for data collection; if/how the instrument(s) changed over the course of the study	Methods>data collection
Units of study - Number and relevant characteristics of participants, documents, or events included in the study; level of participation (could be reported in results)	Results
Data processing - Methods for processing data prior to and during analysis, including transcription, data entry, data management and security, verification of data integrity, data coding, and anonymization/de-identification of excerpts	Methods>data analysis
Data analysis - Process by which inferences, themes, etc., were identified and developed, including the researchers involved in data analysis; usually references a specific paradigm or approach; rationale**	Methods>data analysis
Techniques to enhance trustworthiness - Techniques to enhance trustworthiness and credibility of data analysis (e.g., member checking, audit trail, triangulation); rationale**	Methods>data analysis
Results/findings	

Synthesis and interpretation - Main findings (e.g., interpretations, inferences, and themes); might include development of a theory or model, or integration with prior research or theory	Results
Links to empirical data - Evidence (e.g., quotes, field notes, text excerpts, photographs) to substantiate analytic findings	Results and Codebook
Discussion	
Integration with prior work, implications, transferability, and contribution(s) to the field - Short summary of main findings; explanation of how findings and conclusions connect to, support, elaborate on, or challenge conclusions of earlier scholarship; discussion of scope of application/generalizability; identification of unique contribution(s) to scholarship in a discipline or field	Discussion
Limitations - Trustworthiness and limitations of findings	Limitations
Other	
Conflicts of interest - Potential sources of influence or perceived influence on study conduct and conclusions; how these were managed	Conflict of interest
Funding - Sources of funding and other support; role of funders in data collection, interpretation, and reporting	PlosOne form
