# Supplementary material for: Real-world clinical practice of Diabetic Foot Ulcer prevention and care in Singapore: A qualitative inquiry with healthcare professionals
Source: PLoS One. 2025 Aug 11;20(8):e0328637. doi: 10.1371/journal.pone.0328637 (PMC12338812; doi:10.1371/journal.pone.0328637)
Supplement: S2 Appendix — (PDF) [file pone.0328637.s002.pdf]

S2_Appendix. FGD guide

Theme	Rationale	Guideline referred	Primary question	Secondary question	Further prompt
1. Overview of Work and Lived Experience with DFU care	These questions orient the participants and provide the researcher with an overview of the work that is involved in DFU care.	Constructivist Phenomenological Research Paradigm	What is the scope of your work?	a1. Can you please tell me what a typical day at work looks like for you?<br><br>a2. What is the scope of your work?<br><br>a3. How many DFU patients do you see in a typical week?	What are the tasks that you perform and are responsible for? How many patients do you see a day? Who are the people that you work with most often in your own organization and across agencies?
	These questions enable the participants to reflect on their experience in DFU care, leading to deepened self-understanding, meaning creation, added richness and vividness to the research data.	Eliciting of lived experience of challenges faced and support received in DFU care	What is the most challenging part of your work supporting patients requiring DFU care, and how do you overcome it?	b1. What is the most challenging part of your work supporting patients requiring DFU care? Why?<br><br>b2. How would you overcome such challenges? What support do you need or perhaps have	Challenges in terms of practical skills, knowledge foundation, emotional competence, care coordination, working across agencies, health systems and polices? Support in terms of training, education, teamwork and communication,

				received in the past to overcome these challenges?<br><br>b3. What is the most rewarding parts of your work with DFU patients? Why?	leadership and management, self-care?
	These questions enable the participants to reflect on their experience in working with patients and family carers, leading to deepened self-understanding, meaning creation, added richness and vividness to the research data.	Eliciting of lived experience of working with patients and family caregivers	How is your experience working with DFU patients and family?	c1. How would you describe the relationship between yourself, your team and DFU patients and family carers?<br><br>c2. How would you define good quality care for patients? How about for caregivers?	How do you usually support your patients and caregivers? What are the most common challenges faced in such care partnerships?
2. Foot Assessment	This question helps us to identify what the preferred methods are to detect for neuropathy and PAD.<br><br>Inconsistency in examining for neuropathy and PAD could be the gaps in practice.	"Foot assessment in people with diabetes" guideline by Ministry of Health Singapore suggested the methods to examine deformity, peripheral artery disease (PAD), and neuropathy.<br>Deformity – Look for hammer toe, callus, and hallux valgus<br>PAD – dorsalis pedis, posterior tibial, ABPI, toe pressure<br>Neuropathy – 10 g monofilament as preferred test, 128 Hz tuning fork, neurothesiometer<br><br>Source: Foot assessment in people with diabetes, Agency	How do you look for:<br>a. Loss of protective sensation<br>b. Peripheral artery disease<br><br>What deformities do you look for?	There are several methods to examine for deformity, PAD, and neuropathy.<br><br>a. Which methods do you use to examine the loss of protective sensation?<br>b. What methods do you use to examine for peripheral artery disease?<br>c. Which methods do you use to examine the health of the skin	Which methods do you use to examine for neuropathy and PAD – 10g monofilament, tuning fork, ABPI, toe pressure etc?

		for Care Effectiveness, Ministry of Health Singapore		and toenails of the feet?<br><br>Is there anything else you would like to add?	
3. Prevention of Diabetic Foot Ulcer	Risk stratification is important in DFU prevention. There are multiple guidelines available in guiding risk stratification. This question allows us to identify guidelines adopted by the Singapore specialist care setting. There is a possibility that different settings are adopting different guidelines.	There are multiple guidelines available in guiding risk stratification:<br>I. International Working Group on Diabetic Foot (IWGDF)<br>II. Joint guidelines of the Society for Vascular Surgery, European Society for Vascular Surgery, and World Federation of Vascular Societies<br>I. ADA = American Diabetes Association.<br>II. CDA = Canadian Diabetes Association.<br>III. NHMRC = National Health and Medical Research Council.<br>IV. NWPS = Northwest Podiatry Services.<br>V. OSTEBA = Basque Office for Health Technology Assessment.<br>VI. RNAO = Registered Nurses' Association of Ontario.	How do you prevent and manage diabetic foot ulcers?	We're very interested in the role of specialists' care in relation to the prevention and management of diabetic foot ulcers.<br><br>a. Is there any risk stratification conducted and if yes, how is it done for diabetic foot ulcer in your practice?<br>b. Does your practice(s) follow any specific guidelines?<br>*If yes,*<br>b1. What is(are) the guidelines?<br>*If no,*<br>b2. Why?	What guideline do you use to stratify diabetes patients' risk?

		VII. SIGN = Scottish Intercollegiate Guidelines Network.<br>VIII. SVS = Society for Vascular Surgery. UofA = University of Adelaide.<br><br>In Singapore, appropriate care guide for foot assessment in people with diabetes is available which include risk scarification, referral and patient education.<br><br>Source:<br>I. Screening and Risk Stratification for the Prevention of Diabetic Foot Ulcers: A Summary of Evidence-Based Guidelines (https://www.cadth.ca/sites/default/files/pdf/screening_and_risk_stratification_for_the_prevention_of_DFU.pdf)<br>II. Foot assessment in people with diabetes, Agency for Care Effectiveness, Ministry of Health Singapore			

	This question allows us to understand the screening frequency adopted by the Singapore specialist care setting. This question also allows us to understand if clinicians in the specialist care setting adopted any international guideline in determining patients' screening frequency.	There are several guidelines on screening frequency. For example, based on these risk factors:<br>I. Australian Guidelines on DFU (3 risk groups)<br>II. IWGDF (4 risk groups)<br><br>IWGDF advocates a screening frequency of 1-3 months for people in the highest risk group, compared to 3-6 months in the Australian guideline. However, both acknowledge that this frequency is based predominantly on expert opinion as no evidence is available to indicate superiority of one screening frequency over another.<br><br>Source: Australian and International Guidelines on Diabetic Foot Disease (https://diabeticfootaustralia.org/wp-content/uploads/DFA-Guides-you-through-guidelines.pdf)	How often do you perform foot screening in patients with diabetes?<br><br>Does the screening frequency vary between the risk stratification you shared earlier?	Foot screening frequency in diabetes patients can vary according to different risk groups.<br>c1. How often do you perform foot screening in patients with diabetes?<br>c2. Does the screening frequency vary between the risk stratification you shared earlier?	With what frequency do you prescribe foot assessment to diabetes patients in the low, moderate, and high-risk group?
	This question allows us to understand better on the footwear examination practice in specialist care setting. The lack of footwear examination by podiatrist or clinicians	According to IWGDF guideline, organization of care for diabetic foot disease is important to ensure successful diabetic foot prevention – "Access to measures for reducing risk of foot ulceration, such as podiatric care and	How is footwear examination conducted on patient with diabetes in your clinic?	Could I ask:<br>d1. Is there any footwear examination conducted on patients with diabetes in your clinic?	What is specialist care clinicians' practice on footwear examination?

	can be a gap in the DFU care.	provision of appropriate footwear".<br><br>Source: IWGDF guideline		If yes,<br>d2. Who in the clinic conducts the footwear examination?<br>d3. When is this done?<br>d4. Is there a certain frequency at which this is conducted?<br>*If no,*<br>d5. Why?	
	This question helps to identify the current practice of HbA1c testing in diabetes patients.	Glycaemic control is important in preventing DFU. According to CPG of diabetes, Ministry of Health Singapore, the following schedule is recommended for HbA1c testing in patients with diabetes:<br>• 3-to 4-monthly in patients with unstable glycaemic control, failure to meet treatment goals, recent adjustment in therapy, or intensive insulin therapy.<br>• 6-monthly in patients who have stable glycaemic control and who are meeting treatment goals.<br><br>Source: CPG Diabetes Mellitus, Ministry of Health Singapore	How often do you test the HbA1c in patients with diabetes?	Glycaemic control tends to be important in preventing diabetic foot ulcer.<br>e. How often do you test the HbA1c in patients with diabetes?	What is the frequency of HbA1c test prescribed by specialist care clinicians to diabetes patients at risk for DFU?
	This question aims to explore if clinicians in specialist care tend to engage patients in DFU care. Since we are promoting patient	As suggested by Ministry of Health Singapore, patients should participate in the process of defining their targets of glycaemic control.	What are your patients' role in setting glycaemic control target?	f1. How do you define targets for glycaemic control?<br>f2. Would you involve patients in this process?	What is specialists care clinicians' practice on enhancing patient's empowerment?

	empowerment in DFU care, this question helps in identify gap if patients are less engaged in their DFU treatments.	Source: CPG Diabetes Mellitus, Ministry of Health Singapore		*If yes,*<br>f3. Which patients would you involve in determining the targets of their HbAIc?<br>f4. When would you usually involve them?<br>*If no,*<br>f5. Why?<br><br>Is there anything else you would like to add?	
4. Management of active diabetic foot ulcer	This question allows us to compare the current practice with the guideline	According to the IWGDF guideline, factors that need to be considered in treating and classifying active foot ulcer include:<br>I. Type (neuropathic, neuro-ischaemic, or ischaemic)<br>II. Site and depth<br>III. Cause (footwear)<br>IV. Signs of infection<br>V. Patient related factors (ESRF, oedema, poor metabolic control)<br><br>Based on these factors, there are several classifications system available to determine the severity of diabetic foot ulcers. These include:<br><br>I. Wagner	How do you manage patients with active diabetic foot ulcers?<br><br>What factors would you consider when determining their management plan?	Based on our understanding, you would encounter many patients with active diabetic foot ulcers.<br>a1. How do you manage patients with active diabetic foot ulcers during a typical patient visit? What factors would you consider when determining their management plan?<br><br>a2. Do you use any classification system for diabetic foot ulcers?<br>*If yes,*	What is specialists care practice on the classification and management of active diabetic foot ulcers with multidisciplinary team?

		II. University of Texas<br>III. Site, Ischaemia, Neuropathy, Bacterial Infection and Depth, hence (SINBAD)<br>IV. Perfusion, Extent, Depth, Infection and Sensation (PEDIS)<br>V. ***WIfI Classification System*** (Wound, Ischemia, and foot Infection)<br><br>The most used classification systems are SINBAD, University of Texas and ***WIfI Classification System*** (Wound, Ischemia, and foot Infection. These systems are used to for direct the appropriate ongoing treatment and management plan for diabetic foot ulcer and to predict treatment outcome.<br><br>Studies around the world have shown that setting up an interdisciplinary foot care team (MDT) which include the podiatrists, vascular surgeons, orthopaedic surgeons, endocrinologists, primary care physicians, infectious disease specialists and wound nurse and implementing prevention and management of diabetic foot disease according to the		a3. What are the classification systems used in your setting?<br>*If no,*<br>a4. Why?<br><br>a5. What labs or tests are ordered?<br><br>a6. Do you work with other specialties when providing DFU care?<br><br>a7. If yes, under what circumstances do you refer patients to different services and how is it organised?<br><br>a8. What information is exchanged in this referral? What is the process like?<br><br>a9. Do you encounter the issue of default of scheduled follow-up in patients with DFU?<br><br>a10. What interventions have been conducted in the specialists' care	

		principles outlined in the guideline, is associated with a decrease in the frequency of diabetes related lower-extremity amputations.<br><br>Source: IWGDF guideline; Joint guidelines of the Society for Vascular Surgery, European Society for Vascular Surgery, and World Federation of Vascular Societies		setting to prevent high default rate?<br><br>Is there anything else you would like to add?	
5. Patient Education	This question allows us to understand patient education practice in specialist care. It can be a gap if patient education is not properly conducted in the clinic follow up.	Patient education on diabetic foot care is extremely important in DFU prevention, as IWGDF guideline listed "educating patients, family and healthcare professionals about foot care" as the third "cornerstone of foot ulcer prevention".<br><br>Source: IWGDF guideline	How important is patient education in diabetic foot ulcer prevention?	We would like to talk about patient education now.<br>a. How important is patient education in diabetic foot ulcer prevention?<br><br>a1. What is your view of patient education about caring for diabetic foot ulcers? Is there enough of it? Who does it for your patients?<br>a2. Does the existing patient education work?	What is healthcare practitioners' experience in patient education?<br>Educational interventions for DFU patients (or caregivers)?
	This question allows us to explore the availability of educational materials for patients or caregivers. It could be a potential gap	According to IWGDF guideline, patient's education on diabetic foot care should be presented in a structured, organized, and repeated manner	How is patient education on diabetic foot care delivered at your practice?	B1. Are there any structured educational materials / curriculum pertaining to diabetic	What educational modules or structures for DFU care do you use?

	if there is no 10tandardized educational structure or module in the current practice.	in the prevention of diabetic foot ulcers.<br><br>Source: IWGDF guideline		foot ulcer at your practice?<br>*If yes,*<br>b2. Would it perhaps be possible to share them?<br>*If no,*<br>b3. Could you please share what do they cover?	
	This question allows us to identify the key role person to conduct patient education. It can be a potential gap if there is no health educator in the specialist setting.	According to the IWGDF guideline, a member of the healthcare team should provide structured education individually or in small groups of people, in multiple sessions, with periodical reinforcement, and preferably using a mixture of methods. The structured education should be culturally appropriate, account for gender differences, and align with a patient's health literacy and personal circumstances.<br><br>Source: IWGDF guideline	Does anyone in your practice take on the role of a patient educator? If yes, what does he/she do?	c1. Does anyone in your practice take on the role of a patient educator?<br>*If yes,*<br>c2. What are his / her main functions / responsibilities?<br>*If no,*<br>c3. Why?<br>Is there anything else you would like to add?	Who plays the main role as DFU health educator – wound nurse, clinician, podiatrist?
Others	These questions allow us to understand the strengths and weaknesses of the current DFU care in the specialist setting. The findings can help us to explore the strengths and gaps of the present practice.	N/A	What do you think works well in diabetic foot care in Singapore?<br><br>What do you think could be improved?	Now, we would like to hear your opinion, as a clinician (or wound nurse) in diabetic foot ulcer care:<br>a1. What do you think works well in diabetic foot care in Singapore?<br>a2. Anything else?	What are the strengths and weaknesses of present DFU care in the specialist setting?<br><br>Any suggestions ononon how to improve the current practice in the specialist setting?

	This questions also explore suggestions to improve the present DFU care practice in the specialist care setting from healthcare practitioners' perspective.			b1. Are there any gaps or weaknesses in the health system or level in terms of diabetic foot care?<br>b2. Anything else?<br><br>We are looking at how diabetic foot care could be improved in Singapore.<br><br>c1. What do you think could be improved? Could you share some suggestions from your experience?<br>c2. If you have the opportunity to speak with someone who has the power and resources to better support you in delivering quality care, what would you ask for and advice would you give them?<br>C3. Anything else?	
