# Supplementary material for: Real-world clinical practice of Diabetic Foot Ulcer prevention and care in Singapore: A qualitative inquiry with healthcare professionals
Source: PLoS One. 2025 Aug 11;20(8):e0328637. doi: 10.1371/journal.pone.0328637 (PMC12338812; doi:10.1371/journal.pone.0328637)
Supplement: S3 Appendix — (PDF) [file pone.0328637.s003.pdf]

S3_Appendix: Codebook

Theme	Category	Code	Frequency of the code	Examples of the quotes	ID
Assessment	127				
	Visual inspection	Assessment\Visual inspection	32	Um, we will always check basically at the very least from the knees down, just a check for wounds. And we're looking for any abrasions, any changes in skin, colours, swelling, stuff that's not there before, that's there now, you know, everything and anything, really.	S04P01
	Vascular assessment	Assessment\Vascular assessment	33	Feel for the pulse, dorsalis pedis or PT. If cannot be felt, then we will proceed to use Doppler.	S03P02
	Neurological assessment	Assessment\Neurological assessment	19	So, a quite comprehensive podiatric assessment of the foot will involve monofilament testing at 12 different locations of the feet ranging from the forefoot, the midfoot, to the hindfoot lah.	S01P06
	Footwear	Assessment\Footwear	15	Yes, so, we have, I have the podiatrist with me. We do look at the patient's footwear. We do tell, ask them…. You ask them to bring their usual footwear that they usually wear out. So, more often than not, it's like some Crocs or slippers, which is completely not suitable. So, we do advise them on that.	S04P04
	Wound inspection and progression	Assessment\Wound inspection and progression	14	When we do the wound assessment, we will debride the wound, clean up the wound, and assess the area and the depth lah, and then see whether is there any infection or not and decide the management for that.	S01P01
	Foot shape	Assessment\Foot shape	8	We will check for any deformities like Charcot, or bunions or flat feet, any kind of deformities. So, if there is, we actually will flag to the podiatrist for reviews	S02P02

Theme	Category	Code	Frequency of the code	Examples of the quotes	ID
	Foot temperature	Assessment\Temperature	4	Then subsequently, they also do a temperature check between the two feet to see whether there is any difference of greater than two or three degrees lah, which would signify some degree of inflammation or infection.	S01P06
	Other	Assessment\Other\Medication	2	Check that he's taking the correct medications that I think he should be taking... Change his medications on a computer system, explain to him why he needs a change of medications, explain, perhaps, to next of kin, sometimes in a different language, about why these medication changes that are required.	S03P04
Education	112				
	Formats	40			
		Education\Format\Leaflets	12	So, the nurses have a structured kind of material... With all the info of a patient. Information leaflets and all that, that you share with the patient.	S04P03
				Yeah, we have pamphlets, but I think we can do a lot more, we can probably do a lot more than that.	S03P03
		Education\Format\One-on-one	10	So, the other way we do it is that we spend many clinic consultations to build rapport with the patients, so that eventually the patient understands and trusts our word, then whatever we say they will accept. But that takes a lot of time and that takes many sessions… it doesn't always succeed.	S03P04
		Education\Format\Reinforcement	11	So far, my side on a first screening, right? Normally we will ask for a, a… We will ask patients to relate, what have you learnt previously on the foot screening? So, if they manage to say that we were actually… See physically if they really do it, then from there we go full on.	S02P02
		Education\Format\ Group	2	We also found out that this education is in our skills for life program. So, this skills for life program, usually it's more for patients who are newly diagnosed with diabetes, so they can join this program, so we teach them not only on footcare.	S02P04

Theme	Category	Code	Frequency of the code	Examples of the quotes	ID
		Education\Format\Online	2	If you say if there is any structured curriculum for the patient per se, like, them having to sit down together to go for classes or [get to] e-learn, I think not at this moment, but there is a structure to how we deliver the education to the patient. Yeah. So, the patient [has the access] tothe Health Library, the AA Health Library. They can click and they can read on their own.	S01P04
		Education\Format\Videos	3	Sometimes the patients might wait for a bit longer before they come and see us and then this is the time where we would catch their attention. It really is through the educational videos that we play around in our clinics. I think we're doing quite well with education, education for prevention of disease in Singapore.	S02P01
	Other Aspects	19			
		Education\Other aspects\Caregivers	5	So, basically when we see patients up in the wards when they are about to be discharged, we will usually get their family members or their caregivers to come down to participate in wound dressings.	S02P01
		Education\Other aspects\Patient journey	6	So, I also feel that quality of life, it starts in the start of the diagnosis of the diabetes. But for the DNEs [Diabetes Nurse Educator], the nurses, what we do is when the patient receives diagnosis of diabetes, we will not only go through the type of diabetes that they have, how diabetes will [progress], how the pancreas will [unclear]. We will go through very holistically, even up to the footcare. So, I feel that instilling the knowledge from the start of the diagnosis is very important, so at least they know diabetes is a… somehow is a very complicated disease whereby it affects a lot of the regions in our body. And one of the most commonly affected is the foot, if they don't take care of it well.	S02P04
		Education\Other aspects\Time needed, time spent	6	Our workload is quite high. I don't have much time to sit down with the patient as much as I would like. With the family as much as I would like [clears throat], to, um, do you know, to talk to them and to counsel them.	S04P04

Theme	Category	Code	Frequency of the code	Examples of the quotes	ID
		Education\Other aspects\Topics tailored to the risk stratification	2	So, I would just like to share with you this leaflet that we do with the risk stratification, right? This is actually an improved project that we do. So, before this it was a general foot care education leaflet we would give to all our patients who comes in for diabetes foot screening. So, then we saw that some patients don't really take in the educations or the advice that we give our patients. So then, some of them might throw this away, you know? […] So, basically, it's more targeted I'd say, according to the risk category.	S02P01
	Topics	53			
		Education\Topic\Footwear	16	Then for shoes, advise on covered shoes, not too loose to prevent any friction. Then we advise make sure the soles can be thick enough so it can be cleaned and then inspect the shoes, make sure that there's no sharp edges. And to wear loose garters for the socks, so to not impede any circulation. And also some advices, like when they are outdoors do not walk barefoot. You know, sometimes the HDB [Housing Development Board, public housing in Singapore] blocks that they have some stones they say [when] you step on it, it improves circulation but they end up having bruises or they even have wounds.	S02P04
		Education\Topic\General diabetes education	12	We don't know, you see. But, what they got to know is, what type of food is not so good for them, you see, and also portion control, you know. We tell our patients, you know, it doesn't mean that a cake is bad for you. Yes, please have a cake, but just control the portion, you know. So, you get to enjoy a variety of food. Please go ahead, but just control the portion, and you get to enjoy life at the same time, you know.	S04P03
		Education\Topic\Wounds	8	The education that they get is usually… if you don't fix this, you're going to get an amputation. Because, you know, doctors have gotta do their rounds as fast, as quick…	S04P01

Theme	Category	Code	Frequency of the code	Examples of the quotes	ID
		Education\Topic\Importance of footcare and inspection	5	… inspect your feet every day before you go to bed. So, you can use a mirror or get your partners to help you check the sole of your feet, if there's any cracks or any callus or any corns to do the necessary treatment. So, usually, for corns and calluses, we advise them not to use the metal file, to use the [unclear] the wound and monitor. If it's not getting better, then of course to see the specialist and all.	S02P04
		Education\Topic\Other	4	Don't go for hot water spas. So, some general things that not only maybe they can do at home but for something around the environment that they should also take note of what they can or cannot do. Also, the fish spas,we tell them not to do that. That kind of advice.	S02P04
		Education\Topic\Trimming nails	3	… we'll ask them, okay, cut the nails straight at the corners, don't go into the corner.	S02P03
		Education\Topic\Hygiene (wash, dry)	3	And when they shower, then wipe the wet spaces there, and if they're going to put lotion, they're not to apply it to those spaces.	S02P03
		Education\Topic\Moisturize	2	So, basically we just generally besides inspecting the foot there we just say, so, how your leg you use to putting on moisturiser. So, obviously because they just came back from consultation we saw their skin so scaly and all so obviously we know they are not putting moisturisers enough	S02P04
Guidelines	29				
	Risk Stratification and DFU classification	Guidelines\Risk stratification and DFU classification	10	Yeah, correct. So, with this risk stratification that is in the guideline, right? We came up with this this leaflet. So, this is the, the high-risk, low-risk and moderate risk. So, basically this is given to our patients in diabetes foot screening. When we screen our patients, we stratify them in the risk categories, and we will issue this, this leaflet according to the risk categories of our patients. So, it's more, the education is more directed in that sense.	S02P01

Theme	Category	Code	Frequency of the code	Examples of the quotes	ID
	Specific guidelines	Guidelines\Specific guidelines\IWGDF	5	I believe, we do use the IWDGF more regularly, for our side.	S02P01
		Guidelines\Specific guidelines\ACE	4	So, we follow the ACG Guideline, and previously, we have the TASC Vascular Guidelines as well.	S01P04
		Guidelines\Specific guidelines\Other	3	I mean, I know there are several classification systems, like, the podiatrists use a WIfI score or something like that. I know there's the King's Classification also.	S02P06
	Other Aspects	Guidelines\Other aspects\Not using guidelines	6	Not useful for me... the moment they have a wound, means I have to evaluate and treat. It doesn't matter what's the classification.	S04P04
		Guidelines\Other aspects\Unclear literature	1	And then there's the problem of interpretation, because where I was coming to just now in the barriers, was that some of these patients will have clots in their legs but they are fairly asymptomatic. Whether or not to intervene on these people is actually still controversial in our literature.	S03P04
Referrals	131				
	From	21			
		Referrals\From\Inpatient setting	9	Oh, sure, okay. So, I'm a, er, vascular surgeon. Um, so I will say my, my day-to-day work involves, the morning I will do a round to see all of the patients who are admitted under my care for the various vascular issues. So, which includes diabetic foot disease patients.	S04P04

Theme	Category	Code	Frequency of the code	Examples of the quotes	ID
		Referrals\From\Primary care	6	Number two, another referral source would be patients from the outpatient setting, from the polyclinics and from our GP networks. Which basically find patients with a wound over their legs, and then they get referred over, but not at a stage which is critical enough to go to the emergency department.	S03P03
		Referrals\From\Doctor	3	I think it's quite common that we see patients with Charcot, unfortunately. So, basically… And it's more in multi-medical centre, I don't know why. But usually we got, we get referred when, you know patients are in to see the diabetes doctor. And they come in with very red, hot, swollen foot, and, you know, usually patients claim that, you know, they might have some little traumas in, here or there, so they were referred to us.	S02P01
		Referrals\From\Podiatry	2	Uh, so, for us where S01P06 and I both work, we do have a very robust pathway that the polyclinics actually refer any active DFU to us via what we call the LEAP clinic, which is Lower Limb Amputation Prevention… Lower Extremity Amputation Prevention Programme.	S01P05
		Referrals\From\Specialist	1	Referrals from other specialists within the hospital.	S04P04
	To	47			
		Referrals\To\Podiatrist	16	So, we do, especially for those who have signs of callosities or any deformities, then we'll usually refer them to our podiatrist for review, lah. So I think that's the other big part of the equation which are our podiatry colleagues. So if we do find that any one of them has started to get deformities, or if they certainly have a very clearly clinically obvious, Charcot foot. Then apart from the foot and ankle specialist, we'll get the podiatrist involved for foot care.	S03P03
				If it's the higher-risk ones then, of course, I'll send them to… the podiatrist for a more detailed kind of testing.	S02P06

Theme	Category	Code	Frequency of the code	Examples of the quotes	ID
		Referrals\To\Specialist	13	And we will refer these, we will refer these patients on to, to orthopaedics as soon as possible for further investigation.	S02P01
		Referrals\To\Social	8	Of course, like, behaviour is also another aspect of it, but we tend to see in referral, we tend to see the same patients that are like what S02P01 said… Sometimes they tend to open up a bit more to us, la. So, maybe that why we notice the financial issues is one of the concerns, la.	S02P04
		Referrals\To\Doctor	3	If there's any, like, dryness, any scabs or if the patient, say, doesn't have any moisturiser or there's a new wound develop, of course we will highlight it to the doctors so that they can order maybe the necessary ointments.	S02P04
		Referrals\To\Primary care	2	Um, and then we usually just refer them back to the Polyclinic	S04P04
				So, then what do we do for this group of patients? Uh, I guess that's why I started, you know, delegating the job of keeping a very close eye on these patients to podiatry and primary care setting. And of course, if there is any deterioration, to ask them to refer back to me quickly.	S01P06
		Referrals\To\Psychological	3	…and also, , with regards to patients needing, saysome form of amputation, we engage the help of amputation psychologists as well.	S01P06
		Referrals\To\Inpatient	1	They will immediately be seen in the emergency setting, we will refer them to the emergency department or we will get an immediate referral to be admitted to the ward, right.	S03P04
		Referrals\To\Nurse	1	So, that's why most of the time when people… patient with diabetes, we refer to endocrine, which is the advance practice nurse will come in and speak to them lah, educate them.	S03P02
	Working together	Referrals\Working together	24	Either we call the endocrinologist doctor [to] have a look or… we just drop by the next door the podiatrists there, ask them to do us a favour, come in to see the case. Or sometimes I take a picture, send to Dr X, ask the Dr X for opinion… If sometimes my patient told me they went for, like, foot acupuncture, all this, then usually, I will give them advice, and also, I will drop by the podiatry	S01P03

Theme	Category	Code	Frequency of the code	Examples of the quotes	ID
				room to get some footwear pamphlet given to the patients lah	
	Post-referral appointment-delay	Referrals\Post-referral appointment-delay	39	As I was saying just now, right, obviously, at the start, why are they calling the nurses to go to patients' homes when they have some medical issues, why not for diabetic foot? When, like I say, it's legs that they need to walk with, so it might be very difficult for them to travel frsom home to the centres or to the hospitals? So, that would be, yeah, that would be something to think about, yeah, in my opinion.	S02P03
				It's actually been a very, very good idea, I feel. Because a lot of these patients they have home visits, they have social escorts to bring them to the hospital and everything. So, I think if MOH can just step up and provide even more services for this… This vulnerable group, it will be… It... I think it will really make a difference.	S02P05
				Usually, I, because for me it's better doing an operation than not, because that's my job, right. So, if they have no wounds, um, they have no rest pain, that means they don't have pain in the foot when they're not even moving, um, there's no need for surgery. Er, so actually, we don't really do any, take any, er, further investigations, or evaluation. Um, we just give them some general advice. Um, the podiatrist usually, I run the clinic with a podiatrist. So, the podiatrist will also give them some foot care and footwear advice.<br>Um, and then we usually just refer them back to the Polyclinic,	S04P04
				They are all within the same hospital, so I guess our biggest challenge is getting an appointment, as soon as possible, depending on clinical priority and need. And their clinics, like ours, are always full and the patient [load] is very high.	S03P04

Theme	Category	Code	Frequency of the code	Examples of the quotes	ID
				Compared to, say, constantly I'm just telling a patient, okay, next visit I will book a foot screening for you. And then, next visit comes and there's no slot. And the… health care provider again says, okay, maybe that's fine, we'll wait another three months and see. So, I think it's… It boils down more to the messaging that we are giving, whether it's overt or - whether it's like an unspoken. Kind of implied, yeah.	S02P06
Wound Care	56				
	Medical Procedures	Wound care and caring\Medical procedures	32	We'll do regular wound care, wound management, looking at the psychosocial as well, because you know… if they have any financial issues, then that's when we flag up the social workers so that they can… receive our consumables… that could help with their wound healing	S02P01
	Prescriptions	Wound care and caring\Prescription	24	The podiatrist does give them inner soles, once in a while. You know, if they have normal pressure points. To offload those.<br>We just prescribe them wound sandals, or, you know, those wound shoes which are much more suitable.	S04P04
