# Supplementary material for: Real-world clinical practice of Diabetic Foot Ulcer prevention and care in Singapore: A qualitative inquiry with healthcare professionals
Source: PLoS One. 2025 Aug 11;20(8):e0328637. doi: 10.1371/journal.pone.0328637 (PMC12338812; doi:10.1371/journal.pone.0328637)
Supplement: S4 Appendix — (PDF) [file pone.0328637.s004.pdf]

S4_Appendix: Visualization of the categories and codes

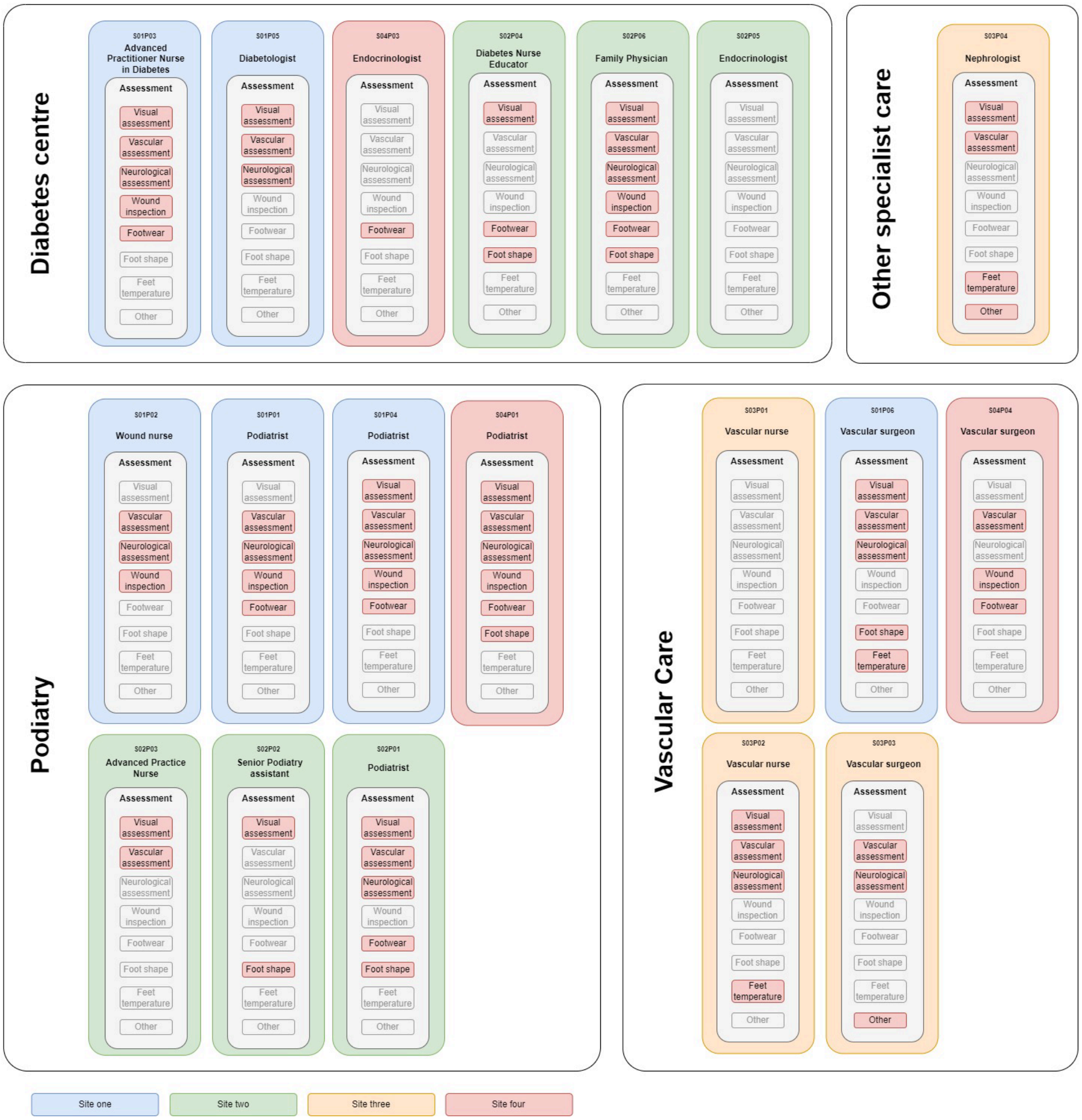

Figure 1. Visual representation of the codes under "assessment" category.

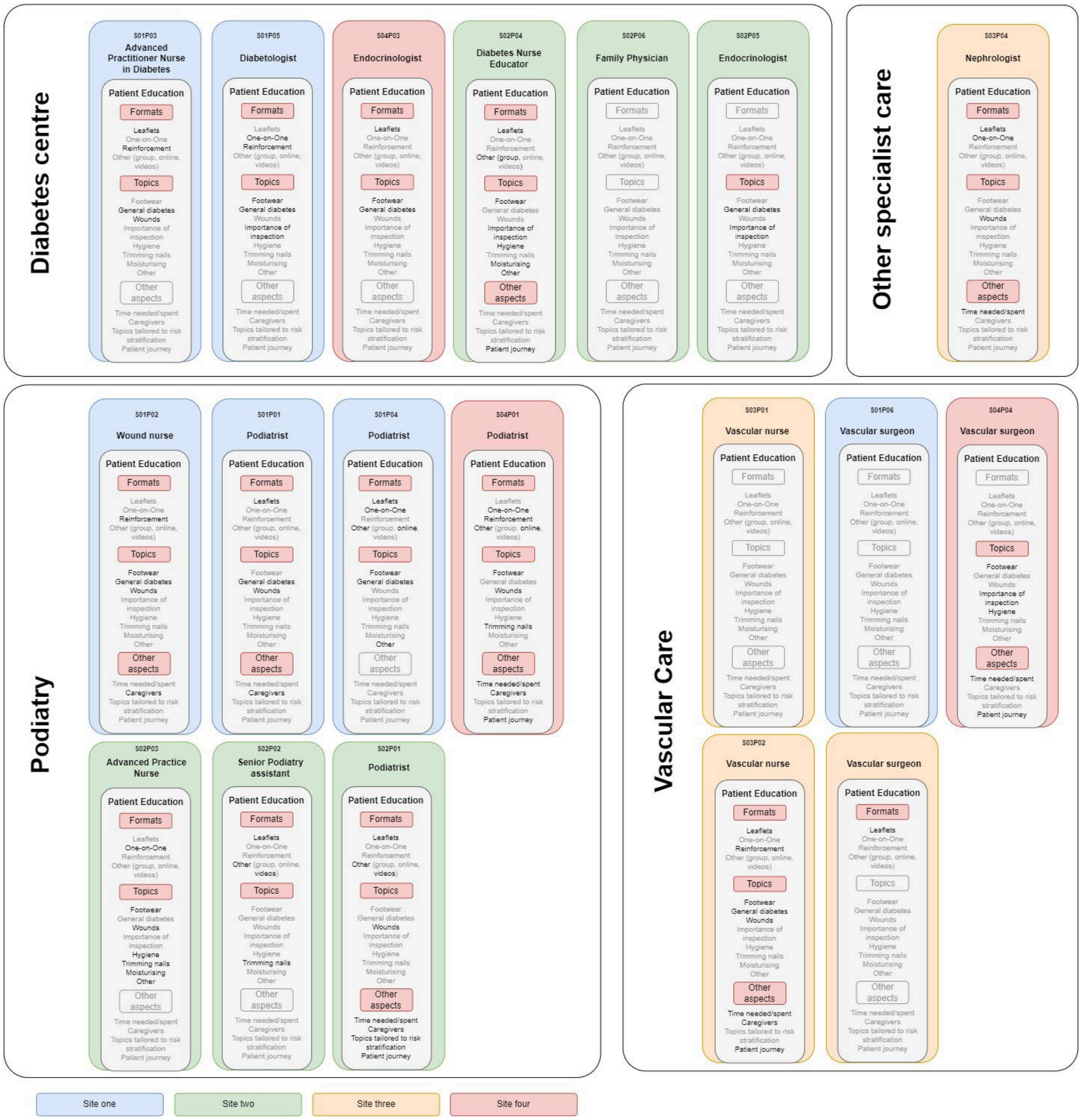

Figure 2. Visual representation of the codes under "patient education" category.

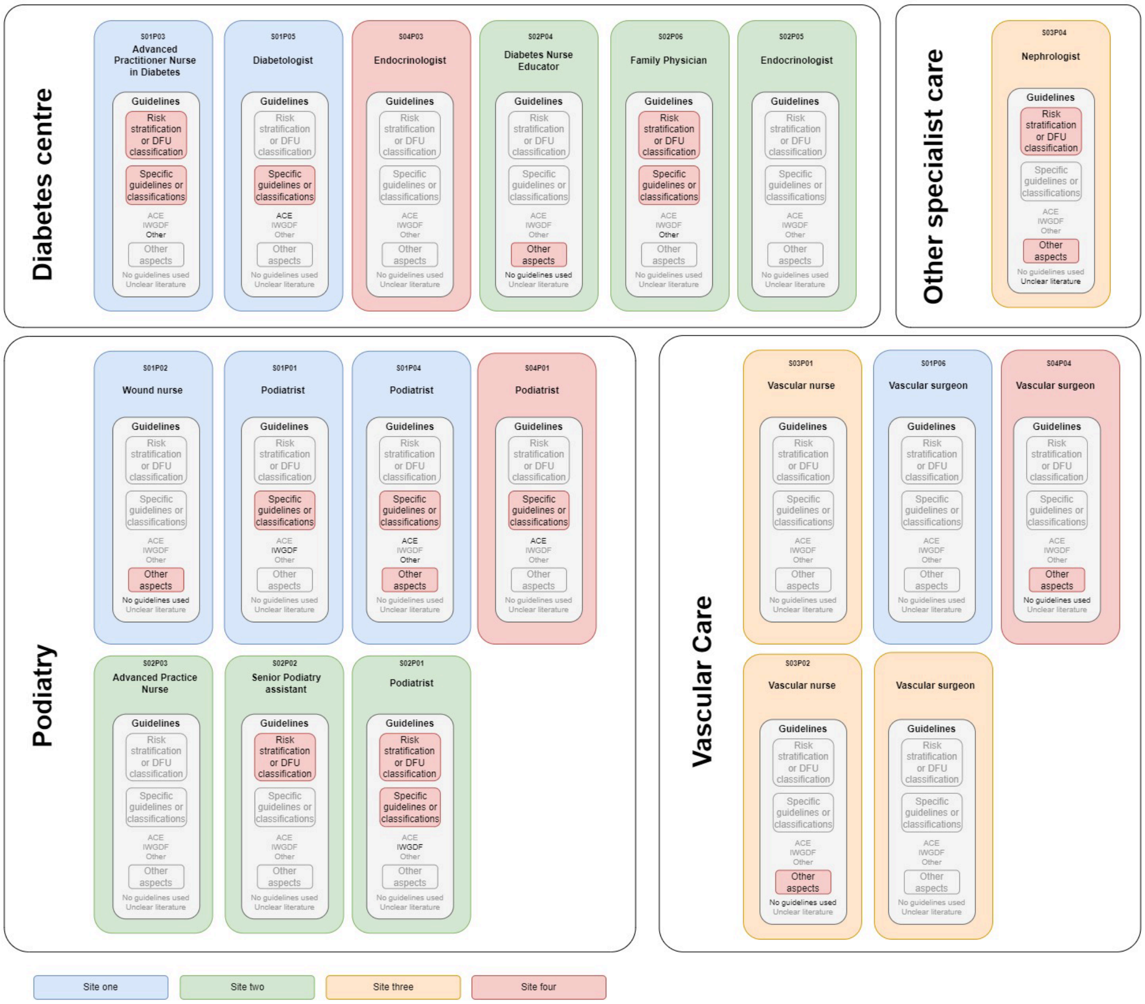

Figure 3. Visual representation of the codes under “guidelines” category.

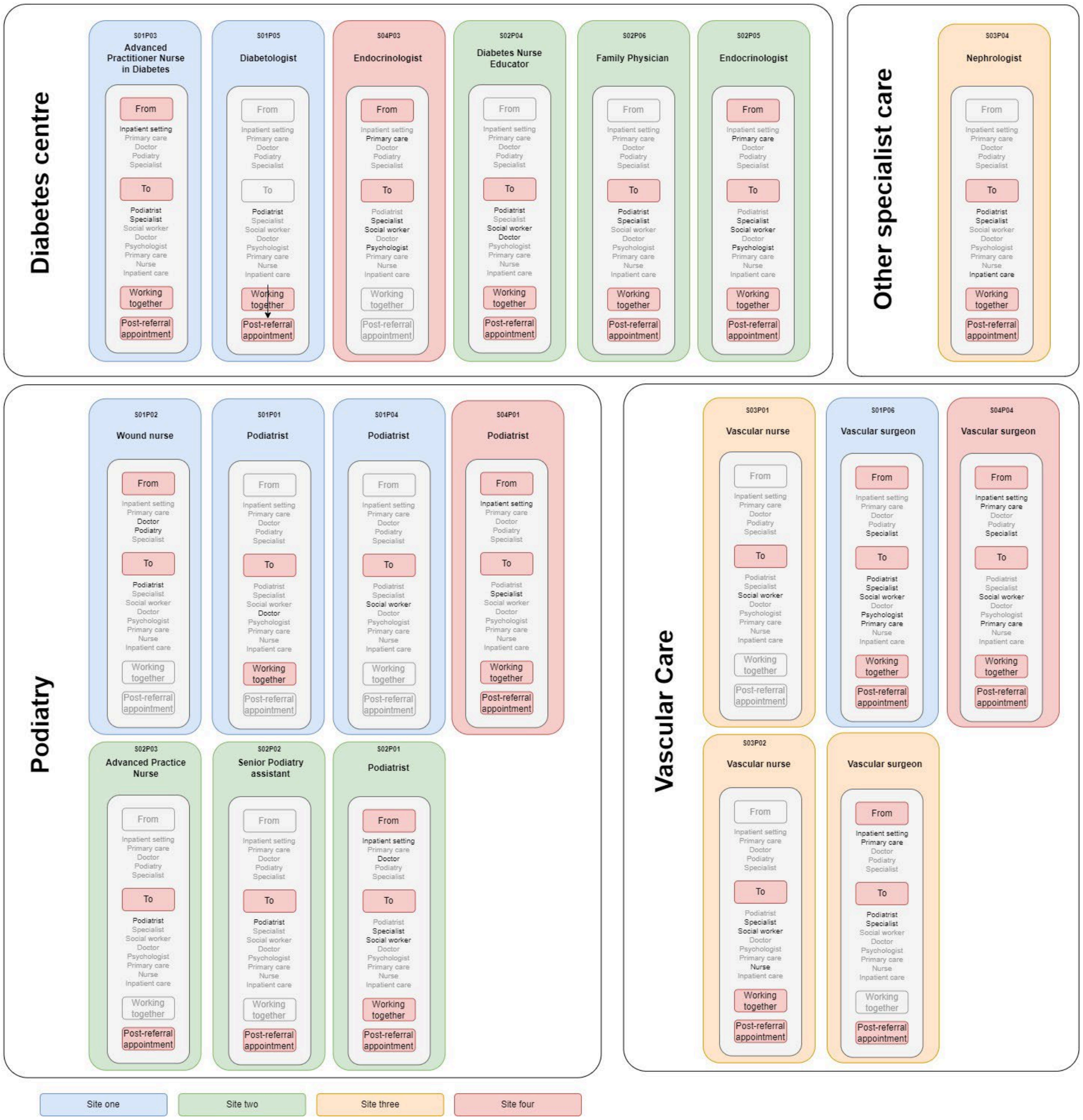

Figure 4. Visual representation of the codes under the “referrals” category.

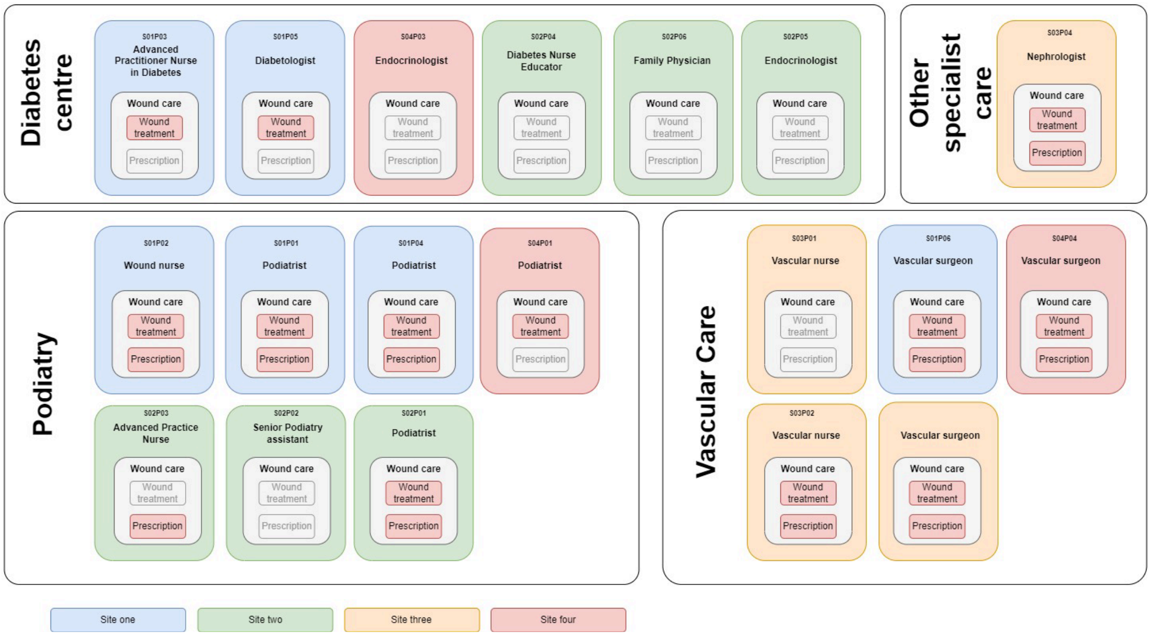

Figure 5. Visual representation of the codes under “wound care” category.
